# *DrugEx* v2: de novo design of drug molecules by Pareto-based multi-objective reinforcement learning in polypharmacology

**DOI:** 10.1186/s13321-021-00561-9

**Published:** 2021-11-12

**Authors:** Xuhan Liu, Kai Ye, Herman W. T. van Vlijmen, Michael T. M. Emmerich, Adriaan P. IJzerman, Gerard J. P. van Westen

**Affiliations:** 1 Drug Discovery and Safety, Leiden Academic Centre for Drug Research, Einsteinweg 55, 2333 CC Leiden, The Netherlands; 2 School of Electronics and Information Engineering, Xi’an Jiaotong University, 28 Xianning W Rd, Xi’an, China; 3 Janssen Pharmaceutica NV, Turnhoutseweg 30, 2340 Beerse, Belgium; 4 Leiden Institute of Advanced Computer Science, Niels Bohrweg 1, 2333 CA Leiden, The Netherlands

**Keywords:** Deep learning, Adenosine receptors, Cheminformatics, Reinforcement learning, Multi-objective optimization, Exploration strategy

## Abstract

In polypharmacology drugs are required to bind to multiple specific targets, for example to enhance efficacy or to reduce resistance formation. Although deep learning has achieved a breakthrough in de novo design in drug discovery, most of its applications only focus on a single drug target to generate drug-like active molecules. However, in reality drug molecules often interact with more than one target which can have desired (polypharmacology) or undesired (toxicity) effects. In a previous study we proposed a new method named *DrugEx* that integrates an exploration strategy into RNN-based reinforcement learning to improve the diversity of the generated molecules. Here, we extended our *DrugEx* algorithm with multi-objective optimization to generate drug-like molecules towards multiple targets or one specific target while avoiding off-targets (the two adenosine receptors, A_1_AR and A_2A_AR, and the potassium ion channel hERG in this study). In our model, we applied an RNN as the *agent* and machine learning predictors as the *environment*. Both the *agent* and the *environment* were pre-trained in advance and then interplayed under a reinforcement learning framework. The concept of evolutionary algorithms was merged into our method such that *crossover* and *mutation* operations were implemented by the same deep learning model as the *agent*. During the training loop, the agent generates a batch of SMILES-based molecules. Subsequently scores for all objectives provided by the *environment* are used to construct Pareto ranks of the generated molecules. For this ranking a non-dominated sorting algorithm and a Tanimoto-based crowding distance algorithm using chemical fingerprints are applied. Here, we adopted GPU acceleration to speed up the process of Pareto optimization. The final reward of each molecule is calculated based on the Pareto ranking with the ranking selection algorithm. The agent is trained under the guidance of the reward to make sure it can generate desired molecules after convergence of the training process. All in all we demonstrate generation of compounds with a diverse predicted selectivity profile towards multiple targets, offering the potential of high efficacy and low toxicity.

**Supplementary Information:**

The online version contains supplementary material available at 10.1186/s13321-021-00561-9.

## Introduction

The ‘one drug, one target, one disease’ paradigm, which has dominated the field of drug discovery for many years, has made great contributions to drug development and the understanding of their molecular mechanisms of action [1]. However, this strategy is encountering problems due to the intrinsic promiscuity of drug molecules, *i.e.* recent studies showed that one drug molecule could interact with six protein targets on average [2]. Side effects of drugs caused by binding to unexpected off-targets are one of the main reasons of clinical failure of drug candidates and even withdrawal of FDA-approved novel drugs [3, 4]. Up to now, more than 500 drugs have been withdrawn from the market due to fatal toxicity [5]. Yet, disease often results from the perturbation of biological systems by multiple genetic and/or environmental factors, thus complex diseases are more likely to require treatment through modulating multiple targets simultaneously. Therefore, it is crucial to shift the drug discovery paradigm to “polypharmacology” for many complex diseases [6, 7].

In polypharmacology, drugs bind to multiple specific targets to enhance efficacy or to reduce resistance formation (in which case multiple targets can be multiple mutants of a single target) [8]. It has been shown that partial inhibition of a small number of targets can be more efficient than the complete inhibition of a single target, especially for complex and multifactorial diseases [6, 9]. In parallel, common structural and functional similarity of proteins results in drugs binding to off-targets. Hence drugs are also required to have a high target selectivity to avoid binding to unwanted target proteins. For example, the adenosine receptors (ARs) are a class of rhodopsin-like G protein-coupled receptors (GPCRs) having adenosine as the endogenous ligand. Adenosine and ARs are ubiquitously distributed throughout human tissues, and their interactions trigger a wide spectrum of physiological and pathological functions. There are four subtypes of ARs, A_1_, A_2A_, A_2B_ and A_3_, each of which has a unique pharmacological profile, tissue distribution, and effector coupling [10, 11]. The complexity of adenosine signaling and the widespread distribution of ARs have always given rise to challenges in developing target-specific drugs [12]. In addition, the similarity to pharmacophores of some generic proteins (*e.g.* the human Ether-à-go-go-Related Gene, hERG) should also be taken into consideration as they can be sensitive to binding exogenous ligands and cause side effects. hERG is the alpha subunit of a potassium ion channel [13] and has an inclination to interact with drug molecules because of its larger inner vestibule as the ligand binding pocket [14]. When hERG is inhibited this may cause long QT syndrome which can be life threatening [15].

In addition to visual recognition, natural language processing, and decision making, deep learning has been increasingly applied in drug discovery [16]. Deep learning does not only perform well in prediction models for virtual screening, but is also used to construct generative models for drug de novo design and/or drug optimization [17]. As an example of the former case our group implemented a fully-connected deep neural network (DNN) to construct a proteochemometric model (PCM) with all high quality ChEMBL data [18] for prediction of ligand bioactivity [19]. Its performance was shown to be better than shallow machine learning methods. Moreover, we also developed a generative model with recurrent neural networks (RNNs), named *DrugEx* for SMILES-based de novo drug design [20]. It was shown that the generated molecules had large diversity and were similar to known ligands to some extent to make sure that reliable and diverse drug candidates can be designed.

Since the first version of *DrugEx* (*v1*) demonstrated effectiveness for designing novel A_2A_AR ligands, we began to extend this method for drug design toward multiple targets. In this study, we updated *DrugEx* to the second version (*v2*) through adding crossover and mutation operations, which were derived from evolutionary algorithms, to the reinforcement learning (RL) framework. We also used Pareto ranking for multi-objective selection. In order to evaluate the performance of our additions we tested our method into both multi-target and target-specific use cases. For the multi-target case, desired molecules should have a high affinity towards both the A_1_AR and A_2A_AR. In the target-specific case, on the other hand, we required molecules to have only high affinity towards the A_2A_AR but a low affinity to the A_1_AR. In order to decrease toxicity and risk of adverse events, molecules were additionally obliged to have a low affinity for hERG in both cases. It is worth noting that generated molecules should also be chemically diverse and have similar physico-chemical properties to known ligands. All python code for this study is freely available at http://github.com/XuhanLiu/DrugEx.

## Materials and methods

### Data source

Drug like molecules represented in SMILES format were downloaded from the ChEMBL database (version 26). After data preprocessing, including standardization of charges, removing metals and small fragments, we collected 1.7 million molecules and named it the *ChEMBL* set, used for SMILES syntax learning. This data preprocessing step was implemented in RDKit [21]. Furthermore, 25,731 ligands were extracted from the ChEMBL database to construct the *LIGAND* set, which had bioactivity measurements towards the human A_1_AR, A_2A_AR, and hERG. The *LIGAND* set was used for constructing prediction models for each target and for fine-tuning the generative models. The number of ligands and bioactivities for these three targets in the *LIGAND* set is represented in Table 1. Duplicate items were removed and if multiple measurements for the same ligands existed, the average pChEMBL value (pX, including pKi, pKd, pIC50, or pEC50) was calculated. To judge if a molecule is active or not, we defined the threshold of bioactivity as pX = 6.5[19]. If pX < 6.5, the compound was predicted as undesired (low affinity to the given target); otherwise, it was regarded as desired (having high affinity).

**Table 1:** The number of ligands and bioactivities for each of the human protein targets A_1_AR, A_2A_AR and hERG in the *LIGAND* set

	A_1_AR	A_2A_AR	hERG
Total ligands	7700	8406	16,733
Bioactivities	13,100	12,129	22,156
Active ligands (pX ≥ 6.5)	1990	2511	924
Inactive ligands (pX < 6.5)	1859	1709	6438
Inactive ligands (No pX)	1764	1993	1275
Other ligands	2087	4704	8906

### Prediction model

In order to predict the pX for each generated molecule for a given target, regression QSAR models were constructed with different machine learning algorithms. To increase the chemical diversity available for the QSAR model we included lower quality data without pChEMBL value, *i.e.* molecules that were labeled as “Not Active” or without a defined pX value. For these data points we defined a pX value of 3.99 (slightly smaller than 4.0) to eliminate the imbalance of the dataset and guarantee the model being able to predict negative samples. During the training process, sample weights for low quality data were set at 0.1, while for data with an exact pX these were set at 1.0. This allowed us to incorporate chemical diversity, while avoiding degradation of model quality. Descriptors used as input were ECFP6 fingerprints [22] with 2048 bits (2048 dimensions, or 2048D) calculated by the RDKit Morgan Fingerprint algorithm (using a three-bond radius). Moreover, the following 19D physico-chemical descriptors were used: molecular weight, logP, number of H bond acceptors and donors, number of rotatable bonds, number of amide bonds, number of bridge head atoms, number of hetero atoms, number of spiro atoms, number of heavy atoms, the fraction of SP3 hybridized carbon atoms, number of aliphatic rings, number of saturated rings, number of total rings, number of aromatic rings, number of heterocycles, number of valence electrons, polar surface area and Wildman-Crippen MR value. Hence, each molecule in the dataset was transformed into a 2067D vector. Before being input into the model, the value of input vectors were normalized to the range of [0, 1] by the MinMax method. Model output value is the probability whether a given chemical compound was active based on this vector.

Four algorithms were benchmarked for QSAR model construction, Random Forest (RF), Support Vector Machine (SVM), Partial Least Squares regression (PLS), and Multi-task Deep Neural Network (MT-DNN). RF, SVM and PLS models were implemented through Scikit-Learn [23], and the MT-DNN model through PyTorch [24]. In the RF, the number of trees was set as 1000 and split criterion was “gini”. In the SVM, a radial basis function (RBF) kernel was used and the parameter space of C and γ were set as [2–5] and [2–15, 25], respectively. In the MT-DNN, the architecture contained three hidden layers activated by a rectified linear unit (ReLU) between input and output layers, and the number of neurons were 2048, 4000, 2000, 1000 and 3 in these subsequent layers. The training process consisted of 100 epochs with 20% of hidden neurons randomly dropped out between each layer. The mean squared error was used to construct the loss function and was optimized by the Adam algorithm [25] with a learning rate of 10^–3^.

### Generative model

As in *DrugEx v1*, we organized the vocabulary for SMILES construction. Each SMILES-format molecule in the *ChEMBL* and *LIGAND* sets was split into a series of tokens. Then all tokens existing in this dataset were collected to construct the SMILES vocabulary. The final vocabulary contained 84 tokens (Additional file 1: Table S1) which were selected and arranged sequentially into valid SMILES sequences through correct grammar.

The RNN model constructed for sequence generation contained six layers: one input layer, one embedding layer, three recurrent layers and one output layer. After being represented by a sequence of tokens, molecules can be received as categorical features by the input layer. In the embedding layer, vocabulary size, and embedding dimension were set to 84 and 128, meaning each token could be transformed into a 128 dimensional vector. For a recurrent layer, the long-short term memory (LSTM) was used as recurrent cell with 512 hidden neurons instead of the gated recurrent unit (GRU) [26] which was employed only in *DrugEx v1*. The output at each position was the probability that determined which token in the vocabulary would be chosen to grow the SMILES string.

During the training process we put a start token (GO) at the beginning of a batch of data as input and an end token (END) at the end of the same batch of data as output. This ensures that our generative network could choose correct tokens each time based on the sequence it had generated previously. A negative log likelihood function was used to construct the loss function to guarantee that the token in the output sequence had the largest probability to be chosen after being trained. In order to optimize the parameters of the model, the Adam algorithm [25] was used for the optimization of the loss function. Here, the learning rate was set at 10^–3^, the batch size was 512, and training steps were set to 1000 epochs.

### Reinforcement learning

**Fig. 1:**
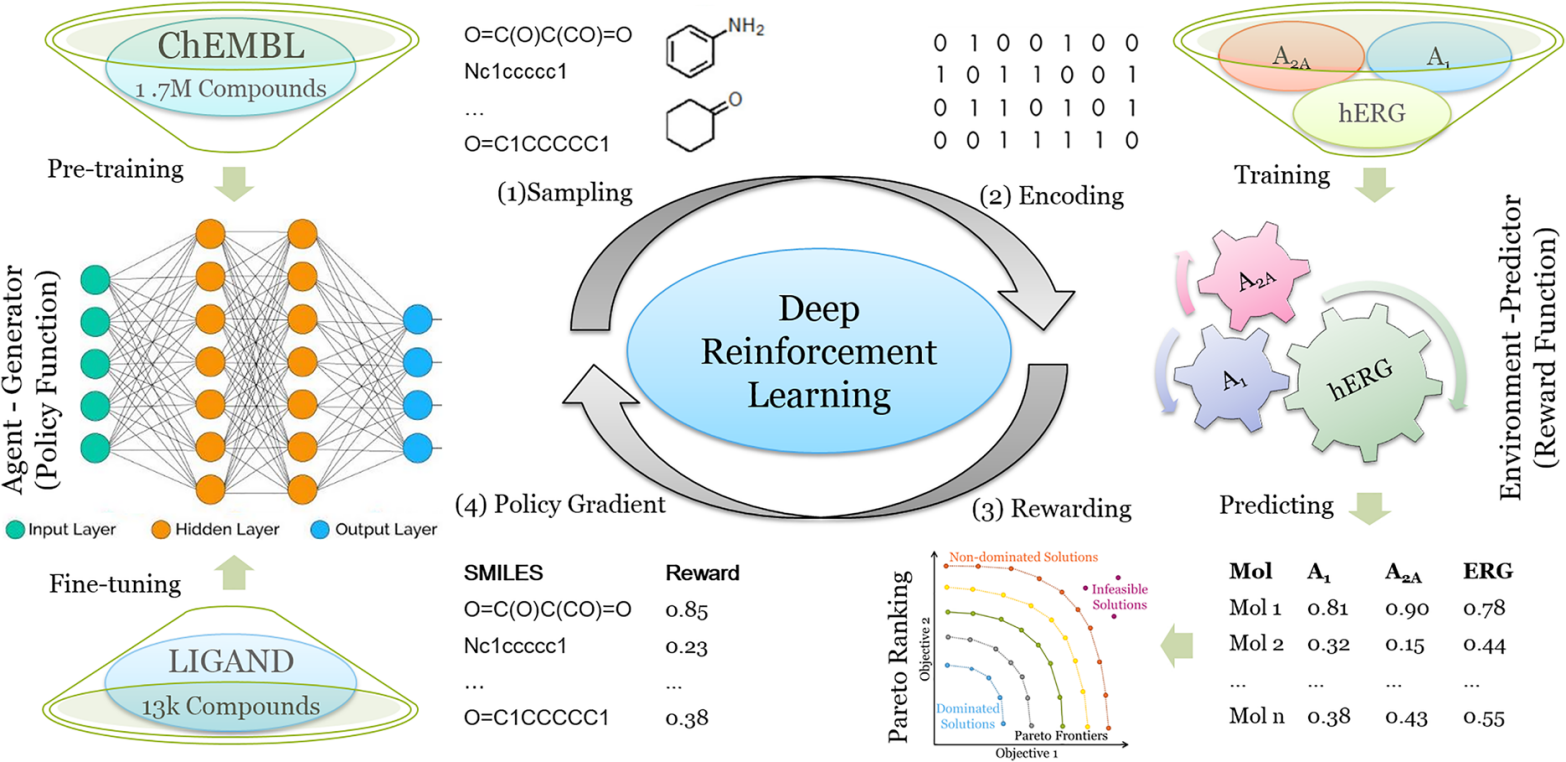
The workflow of the training process of our deep learning-based molecule generator *DrugEx2* utilizing reinforcement learning. After the generator has been pre-trained/fine-tuned, (1) a batch of SMILES are generated by sampling tokens step by step based on the probability calculated by the generator; (2) These valid SMILES are parsed to be molecules and encoded into descriptors to get the predicted pXs with predictors; (3) The predicted pXs are transformed into a single value as the reward for each molecule based on Pareto optimization; (4) These SMILES sequences and their rewards are sent back to the generator for training with policy gradient methods. These four steps constitute the training loop of reinforcement learning

SMILES sequence construction under the RL framework can be viewed as a series of decision-making steps (Fig. 1). The generator (*G*) and the predictors (*Q*) are regarded as the policy and reward function, respectively. In this study we used multi-objective optimization (MOO) and the aim is to maximize each objective for each scenario, albeit with differences in desirability. Our aim was defined by the following problem statement:

$$maximize {R}_{1}, maximize {R}_{2}, \dots , maximize {R}_{n}$$

Here, *n* equals the number of objectives (*n* = 3 in this study), and *R*_*i*_, the score for each objective *i*, was calculated as follows:

$${R}_{i}=\left\{ \begin{array}{l}minmax\left({pX}_{i}\right), \quad\,\,\, if\,high\,affinity\,required\\ {1-minmax(pX}_{i}),\,if\,low\,affinity\,required\\ 0, \quad \qquad\qquad\quad\,\,\, if\,SMILES\,invalid\end{array}\right.$$

Here the *pX*_*i*_ (the range from 3.0 to 10.0) was the prediction score given by each predictor for the *i*^*th*^ target, which was normalized to the interval [0, 1] as the reward score. If having no or low affinity for a target was required (off-target) this score would be subtracted from 1 (inverting it).

For the multi-target case, the objective function is:

$$\left\{\begin{array}{c}{R}_{A1}=minmax\left({pX}_{A1}\right) \\ { {R}_{A2A}=minmax(pX}_{A2A}) \\ \,\,\,\quad{ {R}_{hERG}=1-minmax(pX}_{hERG})\end{array}\right.$$

while the objective function for the target-specific case, is:

$$\left\{\begin{array}{c}\quad\,\,\,\,{R}_{A1}=1-minmax\left({pX}_{A1}\right) \\ { {R}_{A2A}=minmax(pX}_{A2A}) \\ \,\,\,\quad{ {R}_{hERG}=1-minmax(pX}_{hERG})\end{array}\right.$$

In order to evaluate the performance of the generators, three coefficients are calculated with the generated molecules, including validity, desirability, and uniqueness which are defined as:

$$\mathrm{Validity}=\frac{{N}_{valid}}{{N}_{total}}$$

$$\mathrm{Desirability}=\frac{{N}_{desired}}{{N}_{total}}$$

$$\mathrm{Uniqueness}=\frac{{N}_{unique}}{{N}_{total}}$$

where *N*_*total*_ is the total number of molecules, *N*_*valid*_ is the number of the molecules parsed by the valid SMILES sequences, *N*_*unique*_ is the number of molecules which are different from others in the dataset, and *N*_*desired*_ is the number of desired molecules. Here, we determine whether generated molecules are desired based on the reward *R*_*i*_ if all of them are larger than the threshold (0.5 by default when pX = 6.5). In addition, we calculated the SA score (from 1 to 10) for each molecule to measure the synthesizability of which larger value means more difficult to be synthesized [27]. And we also computed the QED (from 0 to 1) score to evaluate the drug-likeness of which larger value means more drug-like for each molecule [28]. The calculation of both SA and QED scores were implemented by RDKit. To orchestrate and combine these different objectives, we compared two different reward schemes: the Pareto front (PF) scheme and the weighted sum (WS) scheme. These were defined as follows:

**Weighted sum (WS) scheme:** the weight for each function is not fixed but dynamic, and depends on the desired ratio for each objective, which is defined as:

$${\mathrm{r}}_{i}=\frac{{N}_{i}^{s}}{{N}_{i}^{l}}$$

Here for objective *i* the *Ns i* and *Nl i* are the number of generated molecules which have a score smaller or larger than the threshold. Moreover, the weight is normalized ratio defined as:

$${w}_{i}=\frac{{r}_{i}}{{\sum }_{k=1}^{M}{r}_{k}}$$

and the final reward R^*^ was calculated by

$${R}^{*}=\sum_{i=1}^{n}{w}_{i}{R}_{i} ,$$

**Pareto front (PF) scheme:** operates on the desirability score, which is defined as

$$ {\text{D}}_{i} = \left\{ {\begin{array}{*{20}c} { 1, \quad if \,R_{i} > t_{i} } \\ { {\raise0.7ex\hbox{${R_{i} }$} \!\mathord{\left/ {\vphantom {{R_{i} } {t_{i} }}}\right.\kern-\nulldelimiterspace} \!\lower0.7ex\hbox{${t_{i} }$}}, \,if \,R_{i} \le t_{i} } \\ \end{array} } \right. $$

where *t*_*i*_ is the threshold of the *i*^*th*^ objective, and we set all of objectives had the same threshold as 0.5 as stated in the methods. Given two solutions *m*_*1*_ and *m*_*2*_ with their scores (*x*_*1*_*, x*_*2*_*, …, x*_*n*_) and (*y*_*1*_*, y*_*2*_,* …, y*_*n*_), then *m*_*1*_ is said to Pareto dominate *m*_*2*_ if and only if:

$$\forall \mathrm{ j}\in \left\{1, \dots ,\mathrm{ n}\right\}:{x}_{j} \ge {y}_{j} \, and \, \exists \mathrm{j}\in \left\{1, \dots ,\mathrm{ n}\right\}:{x}_{j}>{y}_{j}$$

otherwise, *m*_*1*_ and *m*_*2*_ are non-dominated with each other. After the dominance between all pair of solutions being determined, the non-dominated scoring algorithm [29] is exploited to obtain different layers of Pareto frontiers which consist of a set of solutions. The solutions in the top layer are dominated by the other solutions in the lower layer [30]. In order to speed up the non-dominated sorting algorithm, we employed *PyTorch* to implement this procedure with GPU acceleration. After obtaining the frontiers ranking from dominated solutions to dominant solutions, the molecules were ranked based on the average of Tanimoto-distance instead of crowding distance with other molecules in the same frontier, and molecules with larger distances were ranked on the top. The final reward R^*^ is defined as:

$${\mathrm{R}}_{i}^{*}=\left\{\begin{array}{c} 0.5+\frac{k-{N}_{undesired}}{{2N}_{desired}}, \,if\, desired\\ \frac{k}{{2N}_{undesired}}, \qquad\quad\,\,\, if\, undesired\end{array}\right.$$

Here the parameter *k* is the index of the solution in the Pareto rank, and rewards of undesired and desired solutions will be evenly distributed in (0, 0.5] and (0.5, 0.1], respectively.

During the generation process, for each step, *G* determines the probability of each token from the vocabulary to be chosen based on the generated sequence in previous steps. Its parameters are updated by employing a policy gradient based on the expected end reward received from the predictor. The objective function is designated as follows:

$$J\left(\theta \right)={\mathbb{E}}\left[{{R}^{*}(y}_{1:T})|\theta \right]=\sum_{t=1}^{T}logG\left({y}_{t}|{y}_{1:t-1}\right)\cdot {R}^{*}\left({y}_{1:T}\right)$$

By maximizing this function, the parameters $\theta$ in *G* can be optimized to ensure that *G* can construct desired SMILES sequences which can obtain the highest reward scores judged by all the *Qs*.

### Algorithm extrapolation

**Fig. 2:**
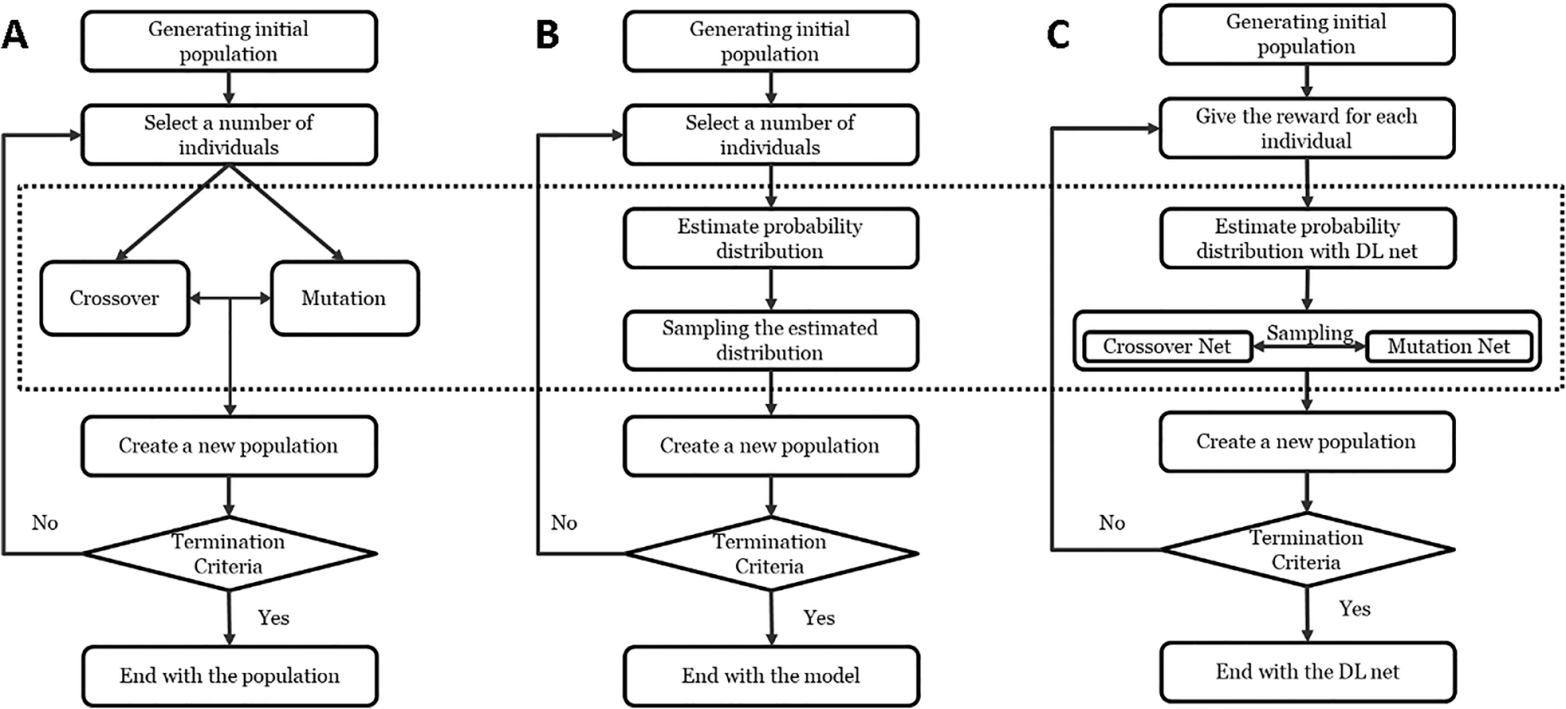
Flowchart comparison of evolutionary algorithms. **A** Estimation of distribution algorithm (**B**) and our proposed method (**C**)

Evolutionary algorithms (EAs) are common methods used in drug discovery [31]. For example, *Molecule Evoluator* is one of EAs, with mutation and crossover operations based on SMILES representation [32] for drug de novo design. In addition, some groups also proposed other variations of EAs [33], e.g., estimation of distribution algorithm (EDA) which is a model-based method and replaces the *mutation* and *crossover* operations with probability distribution estimation and sampling of new individuals (Fig. 2) [34]. Similar to EDA, *DrugEx* is a model-based method too, in which the deep learning model was employed to estimate the probability distribution of sequential decision making. However, we used a DL method to define model-based *mutation* and *crossover* operations. Moreover, we employed an RL method to replace the sample selection step for the update of model or population in EDA or EA, respectively.

### Exploration strategy

**Fig. 3:**
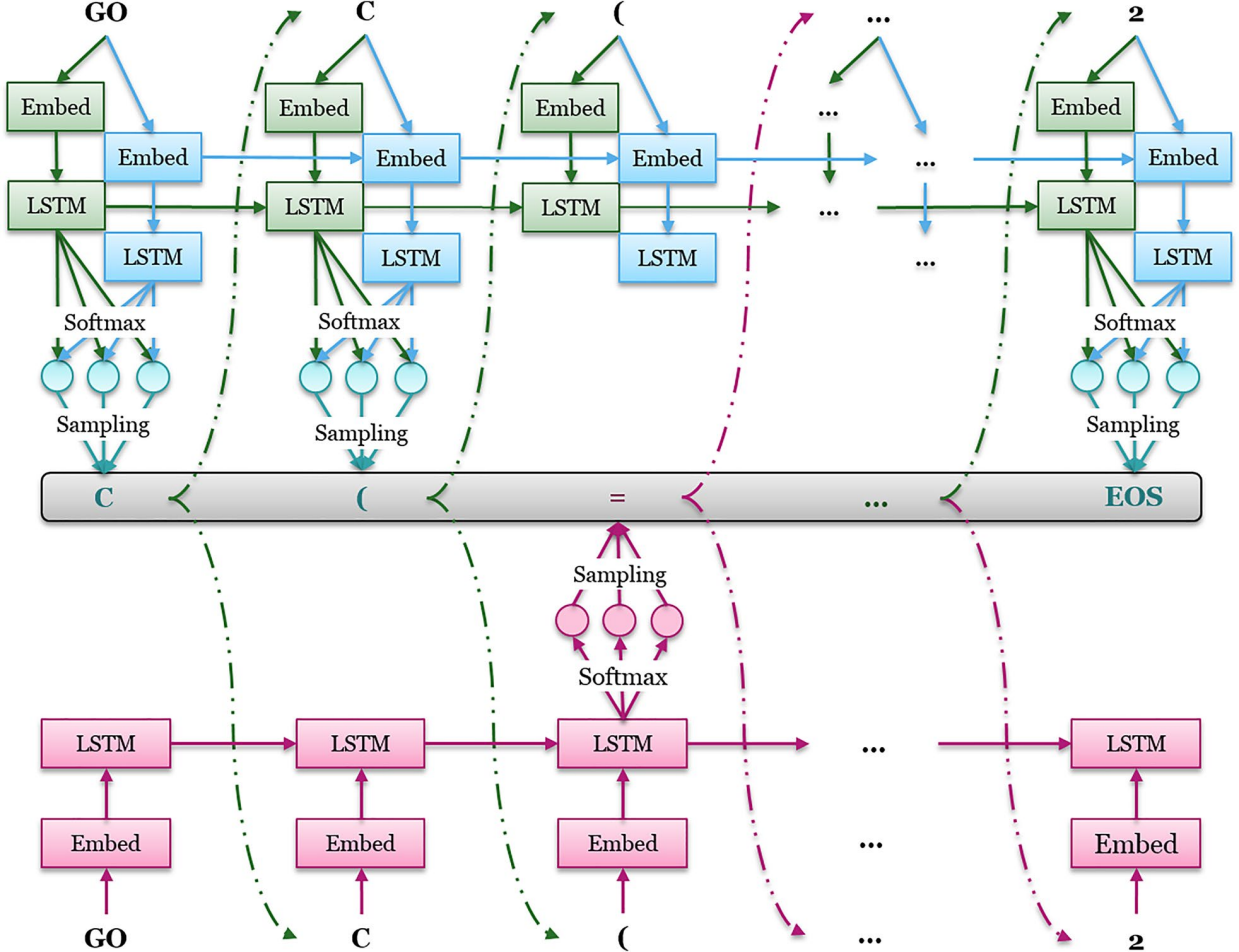
The mechanism of the updated exploration strategy. Shown are the agent net *G*_*A*_, mutation net *G*_*M*_ (red) and crossover net *G*_*C*_ (blue). In the training loop, *G*_*M*_ is fixed, *Gc* is updated iteratively and *G*_*A*_ is trained at each epoch. For each position, a random number from 0 to 1 is generated. If it is larger than the mutation rate (*ε*), the probability for token sampling is controlled by the combination of *G*_*A*_ and *G*_*C*_, otherwise, it is determined by *G*_*M*_

In our previous study, we had implemented the exploration strategy through importing a fixed exploration net to enlarge the diversity of the generated molecules during the training loops. In this study, we continued to extend the methods of this exploration strategy, which resemble the *crossover* and *mutation* operations from evolutionary algorithms (EAs). Here, besides the *agent* net (*G*_*A*_), we also defined exploration strategy with two other DL models: *crossover* net (*G*_*C*_) and *mutation* net (*G*_*M*_), which have the same RNN architecture (Fig. 3). The pseudo code of the exploration strategy is described in Additional file 1: Table S2. Before the training process, *G*_*M*_ was initialized by the pre-trained model while *G*_*A*_ and *G*_*C*_ were started from the fine-tuned model. The *G*_*M*_ was the basic strategy employed in the previous version and its parameters were fixed and not updated during the whole training process. The *G*_*C*_ implemented in this work was an extended strategy whose parameters were updated iteratively based on the G_*A*_. During the training process, each SMILES sequence was generated through combining these three RNNs: for each step, a random number from 0 to 1 is generated. If it is larger than the mutation rate (***ε***), the probability for token sampling is controlled by the combination of *G*_*A*_ and *G*_*C*_, otherwise, it is determined by *G*_*M*_. For each training loop, only the parameters in *G*_*A*_ were updated instantly based on the gradient of the RL objective function. An iteration was defined as the period of epochs after the desirability score of molecules generated by *G*_*A*_ did not increase. Subsequently the parameters of *G*_*C*_ were updated with *G*_*A*_ directly and the training process continued for the next iteration. The training process would continue till the percentage of desired molecules in the current iteration was not better than in the previous iterations.

### Molecular diversity

To measure molecular diversity, we adopted the metric proposed by Solow and Polasky in 1994 to estimate the diversity of a biological population in an eco-system [35]. It has been shown to be an effective method to measure the diversity of drug molecules [36]. The formula to calculate diversity was redefined to normalize the range of values from [1, m] to (0, m] as follows:

$$I\left(A\right)=\frac{1}{\left|A\right|}{{\varvec{e}}}^{\intercal }{F({\varvec{s}})}^{-1}{\varvec{e}}$$

where *A* is a set of drug molecules with a size of *|A|* equal to *m*, ***e*** is an *m*-vector of 1’s and *F(****s****)* = [*f(d*_*ij*_*))*] is a non-singular *m* × *m* distance matrix, in which *f(d*_*ij*_*)* stands for the distance function of each pair of molecule provided as follows:

$$f\left(d\right)={e}^{-\theta {d}_{ij}}$$

here we defined the distance *d*_*ij*_ of molecules *s*_*i*_ and *s*_*j*_ by using the Tanimoto-distance with ECFP6 fingerprints as follows:

$${d}_{ij}=d\left({s}_{i}, {s}_{j}\right)=1-\frac{\left|{s}_{i}\cap {s}_{j}\right|}{\left|{s}_{i}\cup {s}_{j}\right|} ,$$

where | *s*_*i*_ ∩ *s*_*j*_ | represents the number of common fingerprint bits, and | *s*_*i*_ ∪ *s*_*j*_ | is the number of union fingerprint bits.

## Results and discussion

### Performance of predictors

**Fig. 4:**
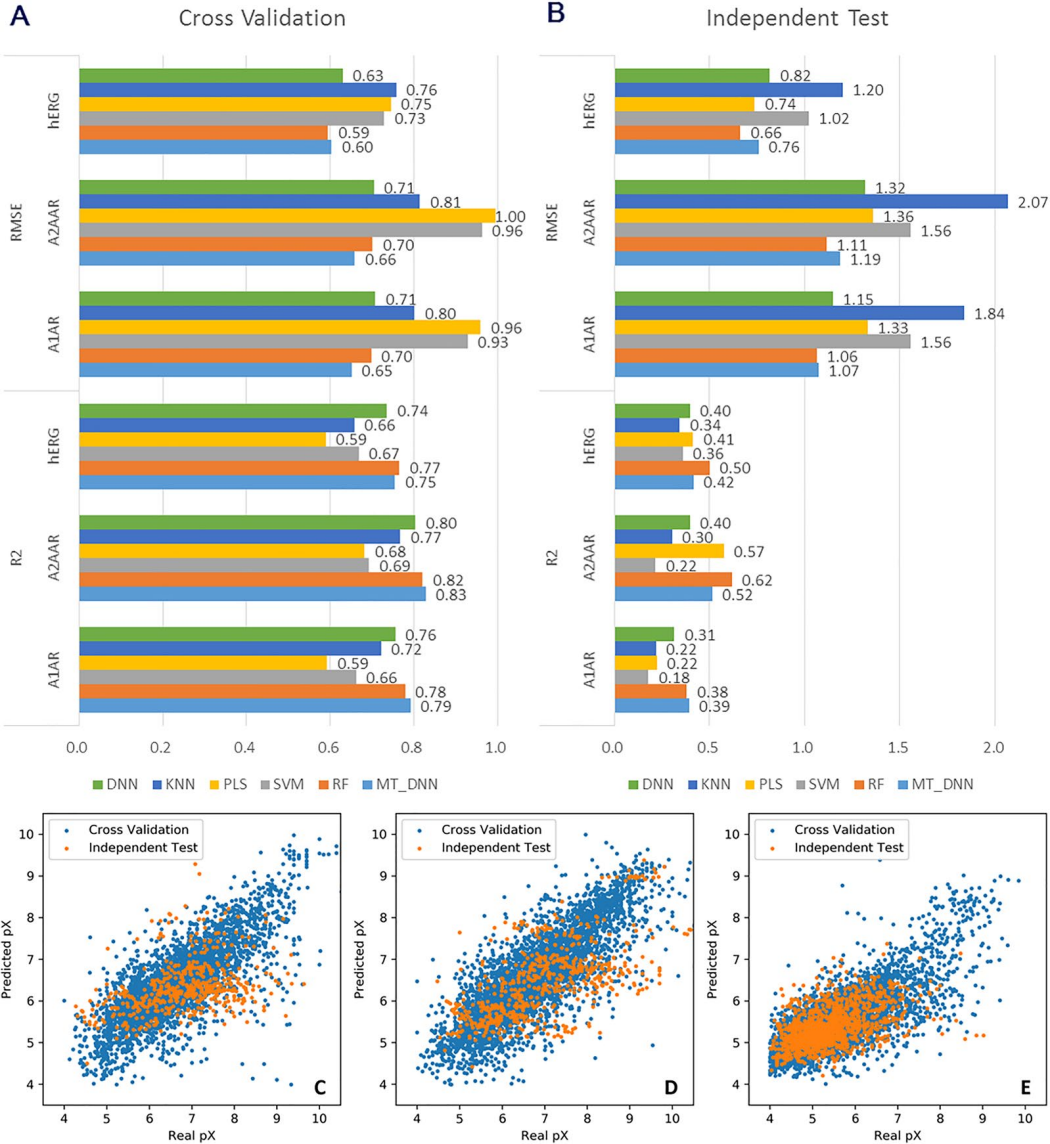
Performance comparison of different machine learning regression models. In these two histograms (**A**, **B**), the results were obtained based on five-fold cross validation (**A**) and independent test (**B**) for the three targets. The R^2^ and RMSE scores were used to evaluate the performance of different machine learning models including DNN, KNN, PLS, SVM RF and MT-DNN. In the scatter plots (**C**–**E**), each point stands for one molecule with its real pX (*x*-axis) and the predicted pX (*y*-axis) by the RF model which was chosen as the final predictors for A_1_AR (**C**), A_2A_AR (**D**) and hERG (**E**) based on five-fold cross validation (blue) and independent test (orange)

All molecules in the *LIGAND* set were used to train the QSAR models, after being transformed into predefined descriptors (2048D ECFP6 fingerprints and 19D physicochemical properties). We then tested the performance of these different algorithms with five-fold cross validation and an independent test of which the performances are shown in Fig. 4A–B. Here, the dataset was randomly split into five folds in cross validation, while a temporal split with a cut-off at the year of 2015 was used for the independent test. In the cross-validation test, the MT-DNN model achieved the highest value for R^2^ and the lowest RMSE value for A_1_AR and A_2A_AR, but the RF model had the best performance for hERG based on R^2^ and RMSE. However, for the independent test the RF model reached the highest R^2^ and lowest RMSE across the board, although it was slightly worse than the performance in the cross-validation test. A detailed performance overview of the RF model is shown in Fig. 4C–E. Because the generative model might create a large number of novel molecules, which would not be similar to the molecules in the training set, we took the robustness of the predictor into consideration. In this situation the temporal split has been shown to be more robust [19, 37]. Hence the RF algorithm was chosen for constructing our environment which provides the final reward to guide the training of the generator in RL.

### Model optimization

As in our previous work in *DrugEx v1*, we firstly pre-trained and fine-tuned the generator with the *ChEMBL* and *LIGAND* set, respectively. When testing the different types of RNNs, we analyzed the performance of the pre-trained model with 10,000 SMILES generated, and found that the LSTM generated more valid SMILES (97.5%) than the GRU (93.1%) which had been adopted in our previous work. Moreover, for the fine-tuning process, we split the *LIGAND* set into two subsets: training set and validation set; the validation set was not involved in parameters updating but it was essential to avoid model overfitting and to improve uniqueness of generated molecules. Subsequently 10,000 SMILES were sampled for performance evaluation. We found that the percentage valid SMILES in fine tuning were again larger for LSTM with 97.9% valid SMILES compared to GRU with 95.7% valid SMILES, a slight improvement compared to the pre-trained model. In the end, we employed the LSTM-based pre-trained/fine-tuned models for the following investigation.

We employed the models for two cases (multi-target and target-specific) of multi-objective drug design towards three protein targets. During the training loop of *DrugEx v2*, the parameter of ***ε*** was set to different values: 10^–2^, 10^–3^, 10^–4^ and we also tested it without the mutation net, *i.e.* the value of ***ε*** was set to 0. Generators were trained by using a policy gradient with two different rewarding schemes. After the training process converged, 10,000 SMILES were generated for each model for performance evaluation. The percentage of valid, desired, unique desired SMILES and the diversity were calculated (Table 2). Furthermore, we also compared the chemical space of these generated molecules with known ligands in the *LIGAND* set. Here, we employed the first two components of a t-SNE of these molecules using ECFP6 descriptors to visualize the chemical space.

**Table 2:** Comparison of the performance of the different methods in the multi-target case

Rewarding scheme	Dataset	Validity	Desirability	Uniqueness	Diversity	Purine ring	Furan ring	Benzene ring
	*LIGAND*	100.00%	12.40%	100.00%	0.66	21.30%	35.44%	79.24%
PF	*DrugEx v1*	98.28%	43.27%	88.96%	0.71	**17.37%**	41.05%	80.95%
	*DrugEx v2*	99.57%	**80.81%**	87.29%	0.7	13.97%	**32.01%**	**80.26%**
	*ORGANIC*	**98.84%**	66.01%	82.67%	0.65	17.27%	56.38%	68.87%
	*REINVENT*	99.54%	57.43%	**98.84%**	**0.77**	0.64%	40.38%	92.05%
WS	*DrugEx v1*	97.76%	38.44%	93.44%	0.71	**10.76%**	**36.42%**	86.99%
	*DrugEx v2*	**99.80%**	**97.45%**	89.08%	0.49	3.63%	21.06%	96.18%
	*ORGANIC*	99.08%	61.10%	77.65%	0.68	9.08%	70.99%	**83.91%**
	*REINVENT*	99.54%	70.98%	**99.11%**	**0.71**	0.04%	23.23%	96.28%

Shown are validity, desirability, uniqueness, and substructure distributions of SMILES generated by four different methods in the multi-target case with PF and WS rewarding schemes. For the validity, desirability and uniqueness, the highest values are bold, while for the distribution of substructures, the bold data are labeled as the closest to the values in the *LIGAND* set

### Performance comparisons

We compared the performance of *DrugEx* *v2* with *DrugEx v1* and two other DL-based de novo drug design methods: *REINVENT* [38] and *ORGANIC* [39]. In order to make a fair benchmark, we trained these four methods with the same environments to provide the unified predicted bioactivity scores for each of the generated molecules. It should be mentioned that these methods are all SMILES-based RNNs generators but trained under different RL frameworks. Therefore, these generators were constructed with the same RNN structures of and initialized with the same pre-trained/fine-tuned models. We also tested *REINVENT* 2.0 [40] but found the training loop did not converge in the PF scheme. We speculate this is due to the number of desired molecules generated by the initial state of the model being too small, not containing enough information. Moreover, addition of a scaffold filter is repetitive when integrated into the PF scheme as it is similar to the similarity-based crowding distance algorithm employed in the PF scheme. Finally, a scaffold filter is a hard condition, because it directly penalizes the score of similar molecules to 0 while the PF scheme decreased the similar molecules. Hence, we have not shown these results here.

In the WS scheme we did not choose fixed weights for objectives but dynamic values which can be adjusted automatically during the training process. The reason for this is that if the fixed weights should be optimized as the hyperparameters, which would be more time consuming. Moreover, the distribution of scores for each objective was not comparable. If the affinity score was required to be higher, few of the molecules generated by the model with the initial state were satisfactory, but if a lower affinity score was required, most of the generated molecules by the pre-trained/fine-tuned model met this need without further training of RL. Therefore, weights were set as dynamic parameters and determined by the ratio between desired and undesired molecules generated by the model at the current training step. This approach ensures that the objectives with lower scores would get more importance than others during the training loop to balance the different objectives and generate more desired molecules.

The performance of the model with different values of ***ε*** is shown in Additional file 1: Table S3. A higher ***ε*** generates molecules with larger diversity but low desirability compared to a lower ***ε*** in both multi-target and target-specific cases. In addition, an appropriate ***ε*** guarantees that the model generates molecules which have a more similar distribution of important substructures with the desired ligands in the *LIGAND* set (Additional file 1: Fig. S1). With the WS scheme, the model generates molecules with a high desirability, but the diversity is lower than the desired ligands in the training set. On the contrary, the PF scheme helped the model generate molecules with a larger diversity than the ligands in the training set, but the desirability was not as high as in the WS rewarding scheme. Importantly, the generated molecules in the PF scheme have a more similar distribution of substructures to the *LIGAND* set than in the WS scheme.

**Fig. 5:**
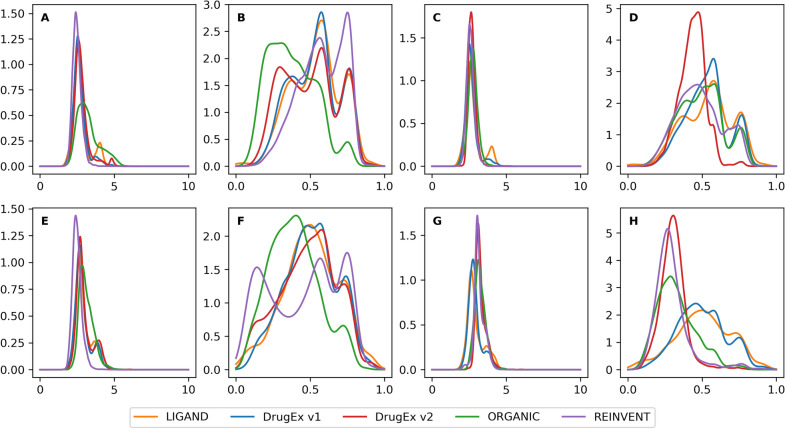
The distribution of SA score and QED score of desired ligands and generated ligands. Shown are the distribution in the *LIGAND* set and of molecules generated by four different methods with PR (**A**, **B**, **E** and **F**) and WS (**C**, **D**, **G** and **H**) rewarding schemes in the multi-target case (**A**–**D**) and target-specific case (**E**–**H**). The molecules from the *LIGAND* set were shown in orange, and the molecules generated by *DrugEx v1, v2, ORGANIC* and *REINVENT* were shown in blue, green, red, and purple, respectively. Overall DrugEx *v1* and *v2* are better able to emulate the observed distributions in the training set compared to *ORGANIC* and *REINVENT*

In the multi-target case, these four methods with different rewarding schemes show similar performance, *i.e.* the WS scheme can help models improve the desirability while the PF scheme assists models to achieve better diversity and distribution of substructures (Table 2). Here, *REINVENT* with the PF scheme achieved the largest diversity, whereas *DrugEx v1* had the most similar substructure distribution to the molecules in the *LIGAND* set, and *DrugEx v2* achieved the best desirability with both PR and WS schemes compared to the three other algorithms. The diversity and distribution of substructures were also most similar to the best results. In addition, in the target-specific case results were similar to the multi-target case, (Table 3), and for the distribution of purine and furan rings, *DrugEx v2* surpassed *v1* to be most similar to the *LIGAND* set. When investigating the SA and QED scores, we observed that the PF scheme helped to make all generated molecules more drug-like because of higher QED scores than the molecules generated by the WS scheme in both multi-target (Fig. 5A–D) and target-specific cases (Fig. 5E–H). Comparing these methods, the molecules generated by *REINVENT* were supposedly easier to synthesize and more drug-like than others, but the molecules of *DrugEx v1* had more similar distributions with the molecules in the *LIGAND* set.

**Table 3:** Comparison of the performance of the different methods in the target-specific case

Rewarding scheme	Dataset	Validity	Desirability	Uniqueness	Diversity	Purine ring	Furan ring	Benzene ring
	*LIGAND*	100.00%	14.63%	100.00%	0.67	28.27%	50.61%	71.84%
PF	*DrugEx v1*	98.07%	48.42%	87.32%	0.73	**29.65%**	61.61%	**70.99%**
	DrugEx v2	99.53%	**89.49%**	90.55%	0.73	23.73%	**56.23%**	67.40%
	*ORGANIC*	98.29%	86.98%	80.30%	0.64	10.60%	89.27%	65.28%
	*REINVENT*	**99.59%**	70.66%	**99.33%**	**0.79**	3.85%	33.82%	92.53%
WS	*DrugEx v1*	97.61%	44.96%	95.89%	**0.68**	78.92%	**80.21%**	68.02%
	*DrugEx v2*	**99.62%**	**97.86%**	90.54%	0.31	19.58%	98.56%	51.87%
	*ORGANIC*	98.97%	88.14%	84.13%	0.49	9.68%%	96.66%	**71.48%**
	*REINVENT*	99.55%	81.27%	98.87%	0.34	**25.13%**	97.52%	74.61%

Shown are validity, desirability, uniqueness, and substructure distributions of SMILES generated by four different methods in the target-specific case with PF and WS rewarding schemes. For the validity, desirability and uniqueness, the highest values are bold, while for the distribution of substructures, the bold data are labeled as the most closed to the values in the *LIGAND* set

**Fig. 6:**
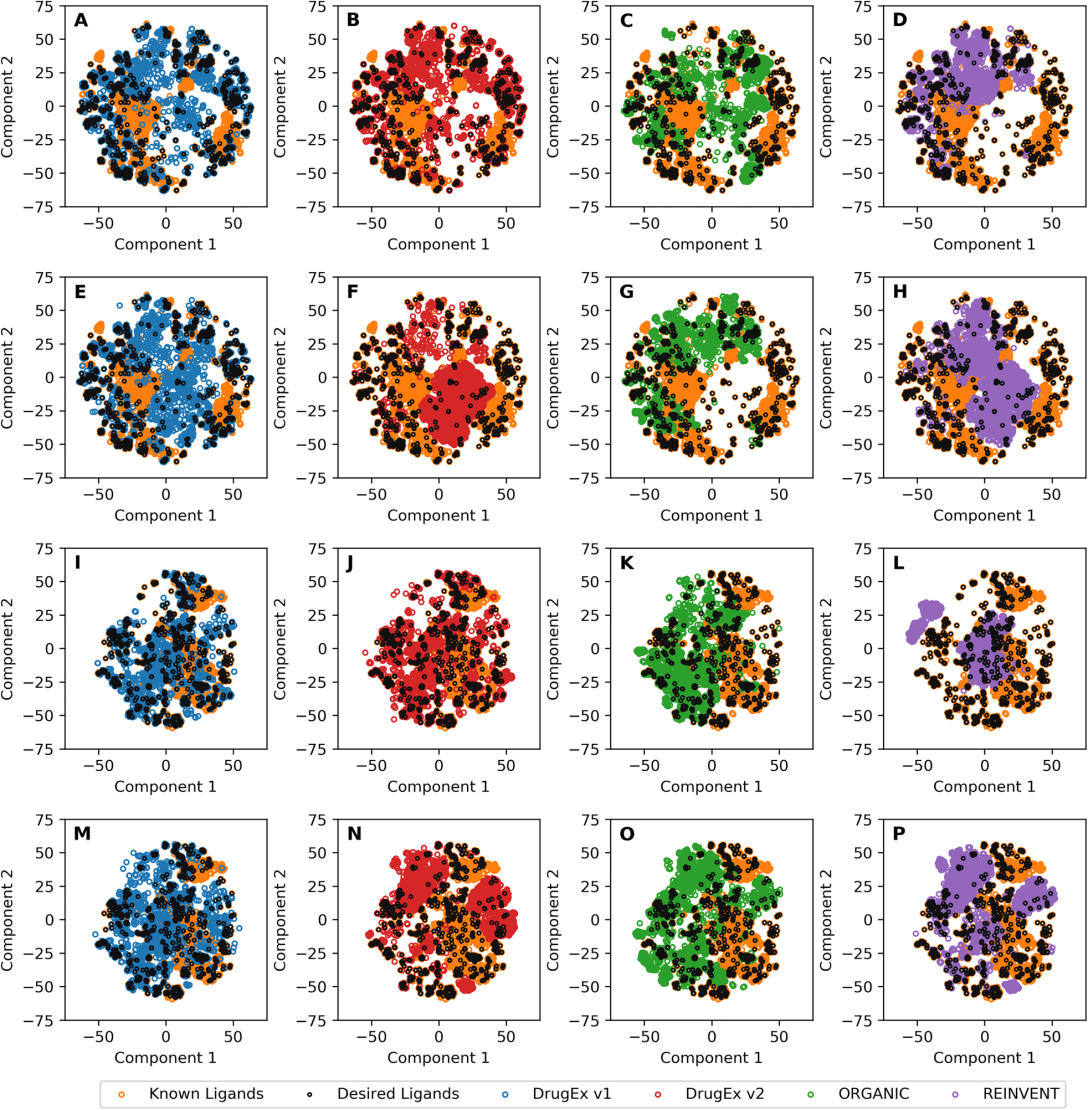
Comparison of the chemical space of the *LIGAND* set and generated molecules. Shown are all known ligands (orange) and desired molecules (black). Moreover shown are generated molecules by *DrugEx v1* (**A**, **E**, **I**, **M**, blue), *v2* (**B**, **F**, **J**, **N**, red)*, ORGANIC* (**C**, **G**, **K**, **O**, green) and *REINVENT* (**D**, **H**, **L**, **P**, purple). A distinction can be made between the multi-target case (**A**–**H**) and target specific case (**I**–**P**). Additionally a distinction can be made between PF scheme based scoring (**A**–**D** and **I**–**L**) and WS scheme based scoring (**E**–**H** and **M**–**P**). Chemical space is represented by the first two components in t-SNE with ECFP6 descriptors of molecules. Similar to our previous work it can be seen that *DrugEx* better covers the whole chemical space of the input data. In particular in the multi-target case with a Pareto optimization based scoring function (**E**–**H**) the improved coverage in all sections, including isolated active ligands, becomes clear

With respect to chemical space, we employed t-SNE with the ECFP6 descriptors of all molecules for both multi-target (Fig. 6A–H) and target-specific cases (Fig. 6I–P). In the multi-target case, most of the desired ligands in the *LIGAND* set were distributed in the margin region of the plot and the PF scheme could guide all of the generators to better cover chemical space than the WS scheme. In the target-specific case, the desired ligands in the *LIGAND* set were distributed more dispersed in both of the margin and the center regions. In this application case only part of the region occupied by desired ligands in the *LIGAND* set overlapped with molecules generated by *REINVENT* and *ORGANIC*. However, almost all of the space is covered by *DrugEx v1* and *v2*. Especially, in contrast to the WS scheme *DrugEx v2* had a significant improvement of chemical space coverage with the PF scheme. Hence in this target-specific case, the PF scheme could not guide all generators for better coverage compared to WS scheme, except for *DrugEx v2*. A possible reason is that the molecules generated by *DrugEx v1* and *v2* offer a more similar distribution of substructures to desired ligands in the *LIGAND* set than *REINVENT* and *ORGANIC* do.

**Fig. 7:**
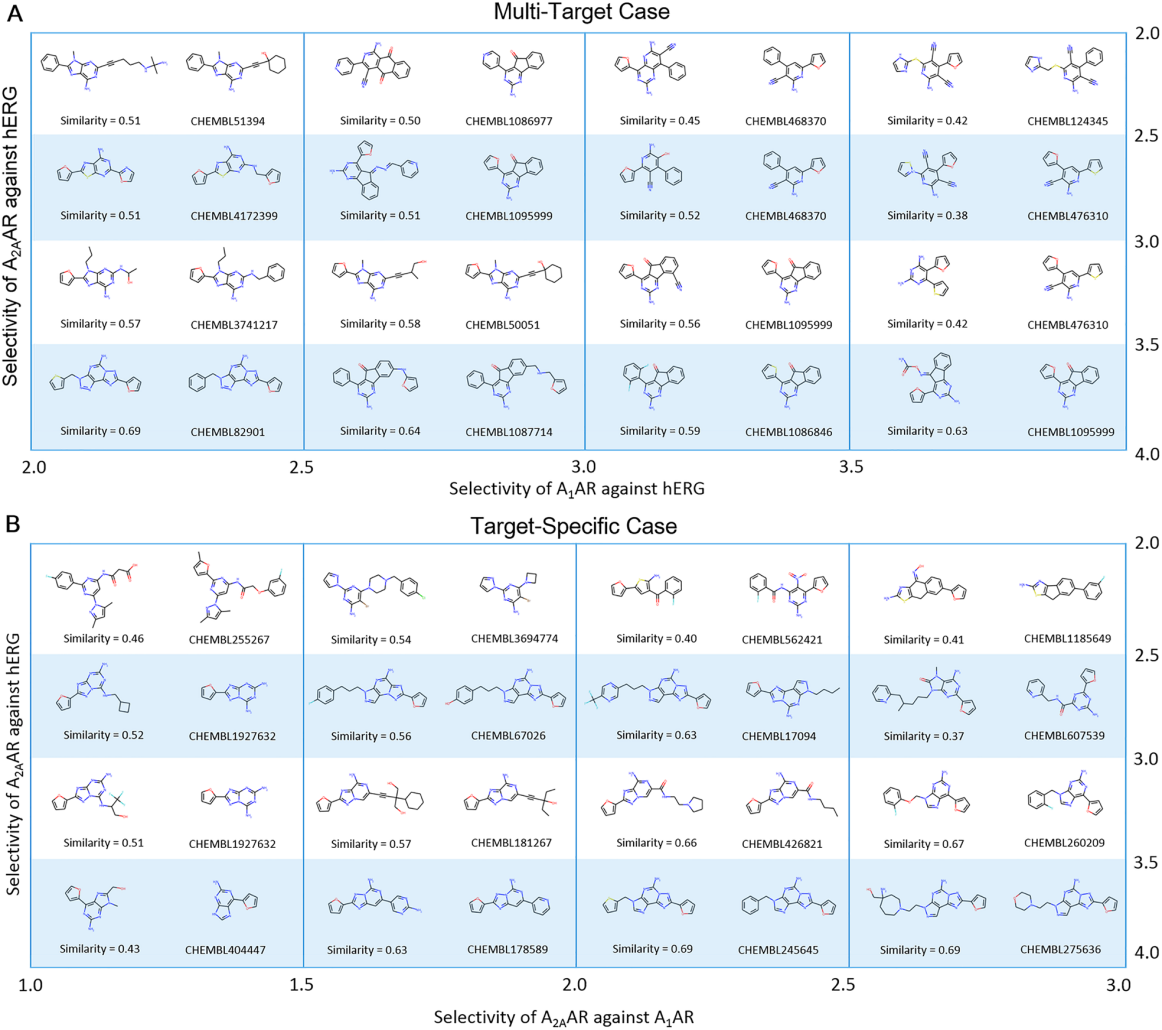
Example molecules generated by *DrugEx v2* with the PF scheme for both multi-target case and target-specific case. In the multi-target case (**A**), these molecules were ordered by selectivity for A_1_AR and A_2A_AR over hERG as *x*-axis and *y*-axis, respectively. In the target-specific case (**B**), these molecules were ordered by selectivity for A_2A_AR over the A_1_AR and hERG as *x* and *y*-axis, respectively. For each cell, the structure at the left is the generated molecule labeled with its similarity to the most similar ligands in the *LIGAND* set, located at the right and labeled with their ChEMBL ID

As an example, 16 possible antagonists (without ribose moiety and with a molecular weight < 500) generated by *DrugEx v2* with the PF scheme were selected as candidates for both multi-target cases and target specific case, respectively. These molecules were ordered by the selectivity which was calculated as the difference of pXs between two different protein targets. In the multi-target case (Fig. 7A) rows and columns are sorted by selectivity for the A_2A_AR and A_1_AR over hERG respectively, because the desired ligands prefer A_1_AR and A_2A_AR to hERG. Conversely in the target-specific case the generated molecules are required to bind only A_2A_AR rather than A_1_AR and hERG (Fig. 7B). Hence, here selectivity for the A_2A_AR over A_1_AR and hERG were represented by the rows and columns respectively.

In order to prove the effectiveness of our proposed method, we tested it with 20 goal-directed molecule generation tasks on the GuacaMol benchmark platform [41]. These tasks contain different requirements, including similarity, physicochemical properties, isomerism, scaffold matching, etc*.* The detailed description of these tasks is provided in ref. [41] and our results are shown in Additional file 1: Table S4. We pre-trained our model with the dataset provided by the GuacaMol platform, in which all molecules from the ChEMBL database are included and similar molecules to the target ligands in the tasks were removed. Then we choose the top 1024 molecules in the training set to fine-tune our model for each task, before reinforcement learning was started. Our method scores the best in 12 out of 20 tasks compared with the baseline models provided by the GuacaMol platform, leading to an overall second place. Moreover, the performance between the LSTM benchmark method and our methods were similar in these tasks, possibly because they have similar architectures of neural networks. All in all, this benchmark demonstrated that our proposed method provides improved performance in de novo design tasks. It is worth mentioning that our method is not effective enough yet for some tasks with contradictory objectives in a narrow chemical space. The main reason is that our method emphasizes to obtain a large number of feasible molecules to cover a diverse chemical space rather than a small number of optimal molecules to achieve the highest score. For example, in the *Sitagliptin MPO task*, the aim is finding molecules which are dissimilar to sitagliptin but have a similar molecular formula to sitagliptin*,* and our method was not as good as Graph GA, which is a graph-based genetic algorithm.

## Conclusions and future prospects

In this work, we proposed a Pareto-based multi-objective learning algorithm for drug de novo design towards multiple targets based on different requirements of affinity scores for multiple targets. We transferred the concept of an evolutionary algorithm (including mutation and crossover operations) into RL to update *DrugEx* for multi-objective optimization. In addition, Pareto ranking algorithms were also integrated into our model to handle the contradictory objectives common in drug discovery and enlarge the chemical diversity. In order to prove the effectiveness, we tested the performance of *DrugEx v2* in both multi-target and target-specific cases. We found that a large percentage of generated SMILES were valid and represented desired molecules without many duplications. Moreover, generated molecules were also similar to known ligands and covered almost every corner of the chemical space that known ligands occupy, which could not be repeated by tested competing methods. In addition to our work here other methods to improve the diversity of generated molecules were proposed such as REINVENT 2.0 [40]. In addition, other teams also trained new deep learning models (e.g. BERT, Transformer, GPT2) with a larger dataset and achieved good results [42, 43]. In future work, we will continue to update *DrugEx* with these new deep learning models to deal with different molecular representations, such as graphs or fragments [31]. We will also integrate more objectives (e.g. stability, synthesizability), especially when these objectives are contradictory, such that the model allows user-defined weights for each objective to generate more reliable candidate ligands and better steer the generative process.

## Supplementary Information

**Additional file 1: Table S1.** All tokens in vocabulary for SMILES sequence construction with RNN model. **Table S2.** The pseudo code of exploration strategy in *DrugEx v2*. **Table S3.** Comparison of validity, desirability, uniqueness and substructures distributions of SMILES generated by *DrugEx v2* with different *ε* in the multi-target and target-specific cases by using PF and WS rewarding schemes, respectively. For the validity, desirability and uniqueness, the largest data is bold, while for the distribution of substructures, the bold data are labeled as the closest to the values in the *LIGAND* set. **Table S4.** Results of the Goal-Directed tasks for our proposed method *DrugEx v2* and other baseline on the GuacaMol Benchmark. GucacaMol platform contains 20 tasks with different requirements, including smilarity, physicochemical properties, isomerism, scaffold matching, etc*.*. The results for baseline models were cited from ref. [41]. The bold data are shown as the best result for each task achieved by different methods. **Figure S1.** Distribution of the SA score and the QED score of desired ligands in the *LIGAND* set and molecules generated by *DrugEx v2.* Shown are distributions with different values of ε in the multi-target case (A-D) and target-specific case (E–H) by using PR (A, B, E and F) and WS (C, D, G and H) rewarding schemes.

## Data Availability

The data used in this study is publicly available ChEMBL data, the algorithm published in this manuscript is made available at https://github.com/XuhanLiu/DrugEx.

## Abbreviations

ARs: Adenosine receptors
DL: Deep learning
MT-DNN: Multi-task deep neural network
ECFP: Extended connectivity fingerprint
EA: Evolutionary algorithm
EDA: Estimation of distribution algorithm
GPCRs: G Protein-coupled receptors
GRU: Gated recurrent unit
LSTM: Long shot-term memory
PF: Pareto front
PLS: Partial least squares
QED: Quantitative estimate of druglikeness
QSAR: Quantitative structure–activity relationship
RBF: Radial basis function
RMSE: Root mean square error
ReLU: Rectified linear unit
RF: Random forest
RL: Reinforcement learning
RNNs: Recurrent neural networks
SA: Synthetic accessibility
SVM: Support vector machine
t-SNE: T-distributed stochastic neighbor embedding
WS: Weighted sum
