# Supplementary material for: DrugEx v2: de novo design of drug molecules by Pareto-based multi-objective reinforcement learning in polypharmacology
Source: J Cheminform. 2021 Nov 12;13:85. doi: 10.1186/s13321-021-00561-9 (PMC8588612; doi:10.1186/s13321-021-00561-9)
Supplement: Supplementary file 1 — Additional file 1: Table S1. All tokens in vocabulary for SMILES sequence construction with RNN model. Table S2. The pseudo code of exploration strategy in DrugEx v2. Table S3. Comparison of validity, desirability, uniqueness and substructures distributions of SMILES generated by DrugEx v2 with different ε in the multi-target and target-specific cases by using PF and WS rewarding schemes, respectively. For the validity, desirability and uniqueness, the largest data is bold, while for the distribution of substructures, the bold data are labeled as the closest to the values in the LIGAND set. Table S4. Results of the Goal-Directed tasks for our proposed method DrugEx v2 and other baseline on the GuacaMol Benchmark. GucacaMol platform contains 20 tasks with different requirements, including smilarity, physicochemical properties, isomerism, scaffold matching, etc.. The results for baseline models were cited from ref. [41]. The bold data are shown as the best result for each task achieved by different methods. Figure S1. Distribution of the SA score and the QED score of desired ligands in the LIGAND set and molecules generated by DrugEx v2. Shown are distributions with different values of ε in the multi-target case (A-D) and target-specific case (E–H) by using PR (A, B, E and F) and WS (C, D, G and H) rewarding schemes. [file 13321_2021_561_MOESM1_ESM.docx]

***DrugEx* v2: *De Novo* Design of Drug Molecules by Pareto-based Multi-Objective Reinforcement Learning in Polypharmacology**

Xuhan Liu^1^, Kai Ye^2^, Herman W. T. van Vlijmen^1,3^, Michael T. M. Emmerich^4^, Adriaan P. IJzerman^1^, Gerard J. P. van Westen^1, *^

^1^Drug Discovery and Safety, Leiden Academic Centre for Drug Research, Einsteinweg 55, 2333 CC, Leiden, The Netherlands

^2^School of electronics and information engineering, Xi’an Jiaotong University, 28 Xianning W Rd, Xi’an, China

^3^Janssen Pharmaceutica NV, Turnhoutseweg 30, B-2340, Beerse, Belgium

^4^Leiden Institute of Advanced Computer Science, Niels Bohrweg 1, 2333 CA, Leiden, The Netherlands

**^*^**To whom correspondence should be addressed: Gerard J. P. van Westen, Drug Discovery and Safety, Leiden Academic Centre for Drug Research, Einsteinweg 55, 2333 CC, Leiden, The Netherlands. Tel: +31-71-527-3511. Email: [gerard@lacdr.leidenuniv.nl](mailto:gerard@lacdr.leidenuniv.nl).

Email Address of other authors: (1) Xuhan Liu: [x.liu@lacdr.leidenuniv.nl](mailto:x.liu@lacdr.leidenuniv.nl); (2) Kai Ye: [kaiye@xjtu.edu.cn](mailto:kaiye@xjtu.edu.cn); (3) Herman W. T. van Vlijmen: [hvvlijme@its.jnj.com](mailto:hvvlijme@its.jnj.com); (4) Michael T. M. Emmerich: m.t.m.emmerich@liacs.leidenuniv.nl; (5) Adriaan P. IJzerman: [ijzerman@lacdr.leidenuniv.nl](mailto:ijzerman@lacdr.leidenuniv.nl).

**Table S1: All tokens in vocabulary for SMILES sequence construction with RNN model.**

| **Atoms** | | | | | | | | **Bonds** | | **Controls** | | | | | |  |
| --- | --- | --- | --- | --- | --- | --- | --- | --- | --- | --- | --- | --- | --- | --- | --- | --- |
| **Common Atoms** | | | | | **Aromatic Atoms** | | | **--** | | **Rings** | | **Branchs** | | **On-Off** | |  |
| B | [As+] | [CH-] | [N] | [SH2] | | [b-] | [se+] | | - | | 1 | | ( | | GO | |
| C | [As] | [CH2] | [O+] | [SH] | | [c+] | [se] | | = | | 2 | | ) | | EOS | |
| F | [B-] | [CH] | [O-] | [Se+] | | [c-] | [te+] | | # | | 3 | |  | |  | |
| I | [BH-] | [I+] | [OH+] | [SeH] | | [cH-] | [te] | |  | | 4 | |  | |  | |
| L | [BH2-] | [IH2] | [O] | [Se] | | [n+] | b | |  | | 5 | |  | |  | |
| N | [BH3-] | [N+] | [P+] | [SiH2] | | [n-] | c | |  | | 6 | |  | |  | |
| O | [B] | [N-] | [PH] | [SiH] | | [nH+] | n | |  | | 7 | |  | |  | |
| P | [C+] | [NH+] | [S+] | [Si] | | [nH] | o | |  | | 8 | |  | |  | |
| R | [C-] | [NH-] | [S-] | [Te] | | [o+] | p | |  | | 9 | |  | |  | |
| S |  | [NH2+] | [SH+] |  | | [s+] | s | |  | |  | |  | |  | |

Considering that the sterochemical information of molecules and ionic bonds were ignored, we removed the “@”, “\”, “/”, “.”.**Table S2: The pseudo code of exploration strategy in** ***DrugEx v2***

| **Algorithm** explore:  **Input:**  G_A_**:** Agent net, G_C_**:** Crossover net, G_M_**:** Mutation net,  ε: mutation rate, size: number of generated molecules  vocab: vocabulary of tokens which is consisted of SMILES sequence.  **Output:**  samples: a list of generated SMILES sequences  samples ← []  **For** i ← 1 to size:  sample ← []  token ← ‘GO’  h ← INIT_STATES ()  mutate ← RANDOM_FLOAT (0, 1)  ratio ← RANDOM_FLOAT (0, 1)  **For** step ← 1 to max_lenth:  prob_A_, h_A_ ← *G_A_* (t, h_A_)  prob_C_, h_C_ ← *G_C_* (t, h_C_)  prob_M_, h_C_ ← *G_M_* (t, h_M_)  **If** ε > mutate **Then**  prob ← prob_M_  **Else**  prob ← prob_A_ * ratio + prob_M_ * ratio  token ← DISTRIBUTION_BASED_SAMPLING (prob, vocab)  **insert** token **to** sample  **If** token == ‘EOS’ **Then**  **Insert** sample **to** samples  **Break**  **End**  **End**  **Return** samples |
| --- |

**Table S3: Comparison of validity, desirability, uniqueness and substructures distributions of SMILES generated by *DrugEx v2* with different *ε* in the multi-target and target-specific cases by using PF and WS rewarding schemes, respectively.** For the validity, desirability and uniqueness, the largest data is bold, while for the distribution of substructures, the bold data are labeled as the most closed to the values in the *LIGAND* set.

| Case | Reward  Scheme | Dataset / ε | Validity | Desirability | Uniqueness | Diversity | Purine Ring | Furan Ring | Benzene Ring |
| --- | --- | --- | --- | --- | --- | --- | --- | --- | --- |
| Multi-Target Case |  | *LIGAND* | 100.00% | 14.63% | 100.00% | 0.67 | 21.30% | 35.44% | 79.24% |
|  | PF | 10^-2^ | 99.39% | 71.37% | **90.47%** | **0.72** | 12.39% | 34.69% | 82.05% |
|  |  | 10^-3^ | 99.57% | 80.81% | 88.96% | 0.71 | **13.97%** | 32.01% | **80.26%** |
|  |  | 10^-4^ | **99.72%** | **83.86%** | 87.19% | 0.71 | 12.45% | 30.58% | 84.04% |
|  |  | 0 | 99.47% | 73.76% | 84.41% | 0.70 | 13.35% | **35.71%** | 81.89% |
|  | WS | 10^-2^ | 99.54% | 87.56% | 93.08% | **0.60** | **9.66%** | **28.83%** | 92.19% |
|  |  | 10^-3^ | **99.80%** | 97.45% | 93.44% | 0.49 | 3.63% | 21.06% | 96.18% |
|  |  | 10^-4^ | 99.79% | **98.15%** | **93.56%** | 0.53 | 2.89% | 24.95% | **91.46%** |
|  |  | 0 | 99.78% | 98.00% | 90.19% | 0.49 | 5.02% | 16.45% | 96.77% |
| Target-Specific Case |  | *LIGAND* | 100.00% | 12.40% | 100.00% | 0.66 | 28.27% | 50.61% | 71.84% |
|  | PF | 10^-2^ | 99.48% | 88.76% | **91.98%** | **0.77** | 18.31% | **47.50%** | 68.95% |
|  |  | 10^-3^ | 99.53% | 89.49% | 87.32% | 0.72 | 23.73% | 56.23% | 67.40% |
|  |  | 10^-4^ | **99.55%** | **91.84%** | 88.31% | 0.74 | **26.86%** | 39.68% | 74.36% |
|  |  | 0 | 99.54% | 91.47% | 88.94% | 0.75 | 22.95% | 43.08% | **71.50%** |
|  | WS | 10^-2^ | 99.16% | 86.45% | 93.97% | **0.42** | **42.84%** | 97.26% | **72.45%** |
|  |  | 10^-3^ | 99.62% | **97.86%** | **95.89%** | 0.31 | 60.81% | 98.56% | 51.87% |
|  |  | 10^-4^ | **99.67%** | 96.82% | 94.56% | 0.34 | 55.14% | **93.69%** | 45.40% |
|  |  | 0 | 99.33% | 96.28% | 92.60% | 0.35 | 42.86% | 98.34% | 63.47% |

**Table S4: Results of the Goal-Directed tasks for our proposed method *DrugEx v2* and other baseline models on GuacaMol Benchmark.** GucacaMol platform contains 20 tasks with different requirements, including smilarity, physicochemical properties, isomerism, scaffold matching, *etc.*. The results for baseline models were cited from ref [41]. The bold data are shown as the best result for each task achieved by different methods.

| Benchmark | Best of Dataset | SMILES GA | Graph MCTS | Graph GA | SMILES LSTM | DrugEx v2 |
| --- | --- | --- | --- | --- | --- | --- |
| Celecoxib rediscovery | 0.505 | 0.732 | 0.355 | **1** | **1** | **1** |
| Troglitazone rediscovery | 0.419 | 0.515 | 0.311 | **1** | **1** | **1** |
| Thiothixene rediscovery | 0.456 | 0.598 | 0.311 | **1** | **1** | **1** |
| Aripiprazole similarity | 0.595 | 0.834 | 0.38 | **1** | **1** | **1** |
| Albuterol similarity | 0.719 | 0.907 | 0.749 | **1** | **1** | **1** |
| Mestranol similarity | 0.629 | 0.79 | 0.402 | **1** | **1** | **1** |
| C11H24 | 0.684 | 0.829 | 0.41 | 0.971 | **0.993** | **0.993** |
| C9H10N2O2PF2Cl | 0.747 | 0.889 | 0.631 | 0.982 | 0.879 | **1** |
| Median molecules 1 | 0.334 | 0.334 | 0.225 | 0.406 | **0.438** | 0.418 |
| Median molecules 2 | 0.351 | 0.38 | 0.17 | 0.432 | 0.422 | **0.435** |
| Osimertinib MPO | 0.839 | 0.886 | 0.784 | 0.953 | 0.907 | **0.967** |
| Fexofenadine MPO | 0.817 | 0.931 | 0.695 | **0.998** | 0.959 | 0.942 |
| Ranolazine MPO | 0.792 | 0.881 | 0.616 | **0.92** | 0.855 | 0.909 |
| Perindopril MPO | 0.575 | 0.661 | 0.385 | 0.792 | 0.808 | **0.812** |
| Amlodipine MPO | 0.696 | 0.722 | 0.533 | 0.894 | 0.894 | **0.898** |
| Sitagliptin MPO | 0.509 | 0.689 | 0.458 | **0.891** | 0.545 | 0.517 |
| Zaleplon MPO | 0.547 | 0.413 | 0.488 | **0.754** | 0.669 | 0.693 |
| Valsartan SMARTS | 0.259 | 0.552 | 0.04 | **0.99** | 0.978 | 0.978 |
| Scaffold Hop | 0.933 | 0.97 | 0.59 | **1** | 0.996 | 0.989 |
| Deco Hop | 0.738 | 0.885 | 0.478 | **1** | 0.998 | 0.986 |
| Total | 12.144 | 14.398 | 9.011 | **17.983** | 17.341 | 17.537 |

**
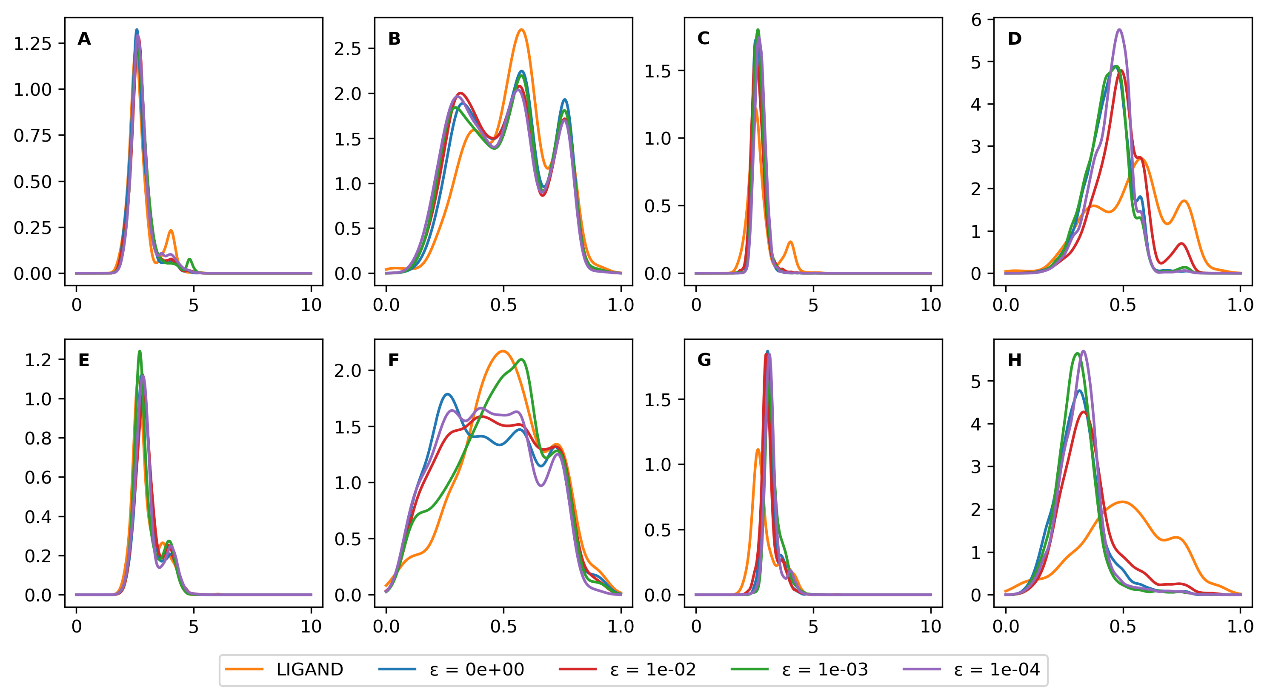
**

**Figure S1: Distribution of the SA score and the QED score of desired ligand in the *LIGAND* set and molecules generated by *DrugEx v2.*** Shown are distributions with different values of ε in the multi-target case (A-D) and target-specific case (E-H) by using PR (A, B, E and F) and WS (C, D, G and H) rewarding schemes.
